# An Analytical Review of Reckless Driving in Ghana: Causes, Consequences and Regulatory Responses

**DOI:** 10.1002/puh2.70320

**Published:** 2026-07-21

**Authors:** Sylvester Kyei‐Gyamfi, Frank Kyei‐Arthur, Marian Kpakpah, Joseph Kwatsenu, Kelvin Aduful, Godwin A. Kubi, Vida Owusua Mensah, Joha Issaka Braimah, Maame Adwoa Nyame Sam, Gloria Ayorkor Charway

**Affiliations:** ^1^ Department of Children Ministry of Gender Children and Social Protection Accra Ghana; ^2^ Department of Environment and Public Health The University of Environment and Sustainable Development Somanya Ghana; ^3^ Ministry of Gender Children and Social Protection Accra Ghana; ^4^ Centre for Migration Studies University of Ghana Accra Ghana

**Keywords:** Ghana, PRISMA guidelines, reckless driving, road safety governance, road traffic injuries, Safe System Approach, traffic enforcement reform

## Abstract

Reckless driving remains a major contributor to road traffic injuries and fatalities in Ghana, posing significant public health and development challenges. This systematic review synthesizes multidisciplinary evidence to examine the multidimensional determinants of reckless driving, its socio‐economic and health consequences and global best practices for sustainable reform. Drawing on the theory of planned behaviour (TPB) and the safe systems approach (SSsA), the study integrates behavioural, institutional, infrastructural and economic perspectives. The findings indicate that speeding, dangerous overtaking, weak enforcement credibility, commercial transport incentive structures and inadequate road design collectively sustain high crash rates. Ghana records thousands of road traffic fatalities annually, with substantial productivity losses and economic costs estimated at 3%–5% of gross domestic product (GDP). Vulnerable road users, particularly pedestrians, bear a disproportionate burden of fatalities. Comparative evidence from countries implementing comprehensive Safe Systems reforms demonstrates that sustained reductions in road deaths are achievable through integrated enforcement, infrastructure redesign, licensing modernization and institutional strengthening. The review concludes that reckless driving in Ghana is preventable through coordinated, evidence‐based policy action and long‐term governance commitment aligned with national development goals.

## Graphical abstract

Reckless driving in Ghana is driven by behavioural norms, economic pressures, weak enforcement and infrastructural deficits. Integrating the theory of planned behaviour and the safe systems approach, this review shows how coordinated reforms, speed management, safer infrastructure, licensing modernization and institutional strengthening can reduce fatalities, improve road safety governance and generate substantial social and economic benefits.

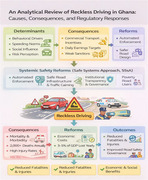

## 1 Introduction

Reckless driving is a major public safety concern globally and remains one of the leading contributors to road traffic crashes, injuries and fatalities. The World Health Organization (WHO) estimates that approximately 1.19 million people die each year due to road traffic injuries (RTIs), making them a leading cause of death worldwide, particularly among young people aged 5–29 years [1]. A significant proportion of these fatalities are linked to unsafe and reckless driving behaviours, which are often categorized as ‘intentional violations’ rather than mere errors in judgement [2].

Reckless driving refers to the operation of a motor vehicle with wilful or wanton disregard for the safety of persons or property. It involves deliberate or negligent behaviours that increase the likelihood of crashes and harm. Globally, reckless driving manifests in several forms, including excessive speeding, dangerous overtaking, driving under the influence of alcohol or drugs, distracted driving, aggressive driving, street racing and disregard for traffic signals [3]. In countries such as the United States, speeding and distracted driving remain major contributors to fatal crashes. Similarly, in India, rapid motorization combined with weak enforcement has intensified road safety challenges. In parts of Brazil and South Africa, aggressive driving, drunk driving and poor compliance with traffic laws continue to fuel high road traffic injury rates. Scholars have noted that these behaviours are frequently exacerbated by ‘optimism bias’, where drivers underestimate the risks associated with their own reckless actions [4].

In sub‐Saharan Africa (SSA), RTIs are particularly alarming. The region records the highest road traffic fatality rates globally, despite having lower levels of motorization compared to high‐income countries [5]. Within this regional context, Ghana has consistently been identified as one of the countries grappling with reckless driver behaviour and rising road crashes. According to the National Road Safety Authority (NRSA), thousands of road crashes are recorded annually in Ghana, resulting in significant injuries and deaths, with human error, particularly speeding, wrongful overtaking and inattentiveness, accounting for the majority of incidents [6]. Research specifically identifies a ‘road safety transition’ in Ghana, where rapid growth in vehicle ownership has not been matched by a corresponding evolution in the regulatory safety culture [7, 8, 9].

Several factors explain why Ghana is frequently noted for reckless driving. First, rapid urbanization and motorization, especially in major cities such as Accra and Kumasi, have outpaced improvements in road infrastructure and traffic management systems. Second, commercial transport operators, particularly drivers of minibuses (trotros) and taxis, often face economic pressures that incentivize speeding and competition for passengers. This ‘commercial pressure’ creates a precarious labour environment where risk‐taking becomes a rationalized survival strategy [10]. Third, weak enforcement of traffic regulations, corruption and inconsistent penalties undermine deterrence. Fourth, inadequate driver training and licensing irregularities contribute to poor driving standards. Finally, risky road‐use culture, where traffic rules are frequently ignored, normalizes unsafe behaviours.

Beyond Ghana, other countries in SSA, including Nigeria and Kenya, also report high levels of reckless driving linked to weak regulatory systems and infrastructural deficits. However, Ghana presents a particularly compelling case for systematic review due to the persistence of road crashes despite ongoing public awareness campaigns and institutional reforms. Although numerous studies and official reports highlight road traffic accidents, there is limited synthesis of the broader determinants, societal consequences and comparative best practices that could inform sustainable reform. The complexity of the Ghanaian context requires moving beyond descriptive statistics toward an understanding of the behavioural and institutional ‘feedback loops’ that sustain reckless driving [11].

This article is justified on several grounds. Although road safety has received policy attention in Ghana, much of the discourse remains fragmented, focusing either on statistical reporting or isolated interventions. There is a need for an integrative and interdisciplinary review that connects behavioural theories, institutional governance, infrastructure deficits and enforcement gaps within a coherent analytical framework. Moreover, Ghana's aspiration toward achieving the Sustainable Development Goals (SDGs), particularly SDG 3.6, which seeks to halve global deaths and injuries from road traffic accidents, demands evidence‐based and context‐specific strategies.

The uniqueness of this article lies in its comprehensive scope and comparative orientation. Rather than merely describing accident trends, it synthesizes global literature, situates Ghana within broader SSA and international contexts and extracts adaptable lessons from countries that have successfully reduced reckless driving through enforcement technologies, public education, graduated licensing systems and road engineering improvements. The integration of the ‘Safe System Approach (SSsA)’, which emphasizes shared responsibility between road users and system designers, serves as the conceptual backbone for these comparisons [12]. By bridging global evidence with local realities, the article provides a strategic roadmap for reform.

The importance of this review extends beyond academia. Reckless driving undermines national productivity, strains healthcare systems, deepens poverty through loss of breadwinners and erodes public trust in regulatory institutions [13, 14]. For policymakers, the article offers a consolidated evidence base to inform legislative reforms, enforcement strengthening and infrastructure investment. For practitioners, including road safety agencies, transport unions and civil society organizations, it provides practical insights into behavioural change strategies and institutional coordination. Ultimately, addressing reckless driving is not merely a transport issue but a public health, development and governance imperative for Ghana. To address these gaps, this review identifies the multidimensional determinants of reckless driving in Ghana, evaluates its socio‐economic and health consequences and analyses global best practices to provide evidence‐based recommendations for sustainable road safety reform.

The remainder of this article is organized as follows. Section 2 presents the theoretical foundation underpinning the study, drawing on the SSsA and the theory of planned behaviour (TPB). Section 3 outlines the systematic review methodology, including the search strategy, inclusion criteria and data synthesis procedures. Section 4 presents the findings on the determinants, consequences and reform options relating to reckless driving in Ghana. Section 5 discusses the findings in relation to existing literature and policy frameworks, whereas Section 5.1 highlights implications for policy and practice. Section 6 discusses the strengths and limitations of the review, and Section 7 concludes with recommendations for sustainable road safety reform in Ghana.

## 2 Theoretical Foundation: SSsA and TPB

This review is anchored in SSsA, complemented by insights from TPB. The integration of these frameworks provides a multi‐level analytical lens consistent with the article's objectives: identifying determinants of reckless driving in Ghana, examining its consequences and synthesizing global reform strategies.

The SSsA, widely adopted in countries such as Sweden and Australia, is premised on the idea that road traffic deaths are preventable and that responsibility for safety is shared among road users, policymakers, vehicle manufacturers and infrastructure designers. Rather than attributing crashes solely to individual ‘human error’, the framework emphasizes system design, safer roads, safer vehicles, safer speeds and effective post‐crash response. This approach aligns strongly with the article's second and third objectives: assessing consequences and examining best practices for reform. It enables the review to move beyond blaming drivers toward analysing institutional weaknesses, enforcement gaps and infrastructural deficits within Ghana's road safety regime.

However, the SSsA has limitations. It may underemphasize micro‐level psychological drivers such as risk perception, social norms and economic pressures influencing commercial drivers. It can also be resource‐intensive, requiring advanced infrastructure and enforcement technologies that may pose fiscal constraints in lower middle‐income contexts, like Ghana.

To address these gaps, the TPB is incorporated. TPB posits that behaviour is shaped by attitudes, subjective norms, and perceived behavioural control. In the Ghanaian context, reckless driving may be normalized through peer influence, commercial competition, weak sanctions and optimism bias. TPB therefore strengthens the first objective by offering a behavioural explanation for speeding, dangerous overtaking and disregard for traffic rules. It helps explain how economic precarity and weak regulatory credibility shape drivers’ intentions and risk‐taking decisions.

Nonetheless, TPB has weaknesses. It assumes rational decision‐making and may inadequately capture structural constraints, such as poor road engineering or corruption in enforcement systems. It also focuses primarily on individual agency, potentially overlooking institutional and political dynamics.

The combined application of the SSsA and TPB offers conceptual complementarity. Although TPB explains why drivers engage in reckless behaviour, the SSsA explains how systemic reforms can mitigate risk even when human error persists. Together, they provide a robust theoretical fit for this review by bridging behavioural, structural and policy dimensions. This integrative framework enhances the article's analytical depth, supports comparative synthesis of global evidence and strengthens its policy relevance for sustainable road safety reform in Ghana.

To operationalize this conceptual integration, this study develops a theoretical framework that links behavioural drivers of reckless driving with systemic safety interventions within Ghana's road transport environment. The framework conceptualizes reckless driving as the outcome of interacting behavioural intentions and structural conditions. Drawing from TPB, driver behaviour is influenced by attitudes toward risk‐taking (e.g., acceptance of speeding), subjective norms within commercial driving communities (e.g., competitive passenger acquisition) and perceived behavioural control shaped by enforcement credibility and regulatory oversight. These behavioural determinants interact with structural conditions emphasized by the SSsA, including road infrastructure design, speed management systems, licensing and driver training regimes, institutional governance and post‐crash response capacity.

Within this integrated framework, risky driving behaviours increase crash probability, whereas systemic weaknesses amplify crash severity. Conversely, improvements in infrastructure safety, enforcement reliability, licensing systems and trauma response mechanisms reduce both the likelihood and consequences of RTIs. The framework, therefore, positions reckless driving not as an isolated behavioural problem but as the product of interconnected behavioural and institutional dynamics.

**FIGURE 1:**
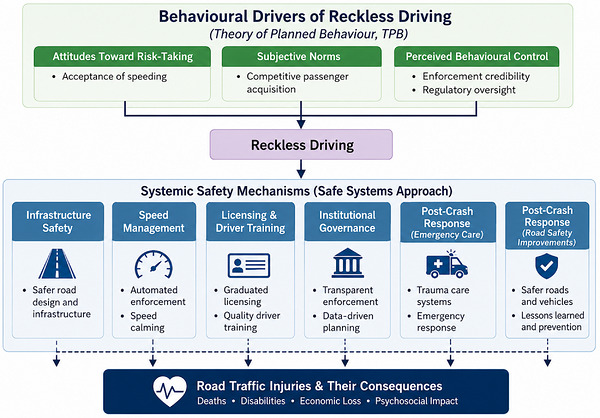
Integrated Behavioural–Systemic Theoretical Framework for understanding and preventing reckless driving in Ghana (TPB–SSsA Integration).

Figure 1 presents the Integrated Behavioural–Systemic Road Safety Framework developed for this review. The figure illustrates how behavioural determinants derived from TPB interact with systemic safety mechanisms emphasized in the SSsA to influence road traffic outcomes in Ghana. By visually integrating these theoretical components, the framework highlights the need for simultaneous behavioural change and institutional reform to achieve sustainable reductions in road traffic fatalities.

The framework (Figure 1) strengthens the analytical foundation of the study by clarifying the causal pathways linking driver decision‐making, institutional governance, infrastructure design and road safety outcomes. It also provides a conceptual bridge between the empirical findings presented in Section 4 and the reform strategies discussed later in the article. By demonstrating how behavioural motivations and systemic safeguards interact, the framework underscores the central argument of this review: effective road safety reform in Ghana requires coordinated interventions that address both driver behaviour and structural safety systems simultaneously.

## 3 Methods

### 3.1 Research Design

This study adopts a systematic review design to synthesize empirical, institutional and policy evidence on reckless driving in Ghana and comparative road safety reform strategies. A systematic review approach was selected because it enables transparent, structured and replicable integration of fragmented evidence across disciplines [15, 16]. Road safety research spans public health, transport engineering, behavioural psychology, economics and governance studies; therefore, systematic synthesis is appropriate for consolidating heterogeneous findings into a coherent analytical framework [17, 18].

The review was guided by the Preferred Reporting Items for Systematic Reviews and Meta‐Analyses (PRISMA) reporting standards to ensure methodological transparency across identification, screening, eligibility and inclusion stages [19, 20]. Systematic reviews are widely recommended in injury prevention and road safety research to strengthen evidence‐informed policymaking and reduce selective citation bias [1, 21].

Consistent with the study objectives, (i) identifying determinants of reckless driving in Ghana, (ii) examining socio‐economic and public health consequences and (iii) analysing global best practices for reform, the design integrates quantitative crash analyses, behavioural surveys, enforcement evaluations, governance diagnostics and comparative policy assessments. Both peer‐reviewed studies and methodologically transparent institutional reports were included, consistent with best practice in transport and safety policy reviews [17, 18].

Given the diversity of evidence sources and outcome measures, the review was designed primarily as a systematic narrative review rather than a quantitative meta‐analysis. The objective was to provide a structured and transparent synthesis of available evidence rather than generate pooled effect estimates.

### 3.2 Scope and Context

The primary geographical focus of the review is Ghana, situated within the broader SSA road safety landscape. SSA records the highest road traffic fatality rates globally relative to motorization levels [5]. Ghana presents a compelling case due to persistent crash prevalence despite institutional reforms and public awareness campaigns led by the NRSA [6].

Comparative evidence from selected SSA countries (e.g., Nigeria and Kenya) and high‐income reform leaders such as Sweden and Australia was incorporated to facilitate benchmarking under the Safe Systems framework [12, 22]. Including both domestic and comparative evidence enhances contextual sensitivity while enabling structured cross‐country learning.

### 3.3 Search Strategy

A structured literature search was conducted between January and April 2025. Multiple electronic databases were searched to enhance comprehensiveness and reduce publication bias, consistent with systematic review standards [20, 23]. The databases included the following:

- Scopus
- Web of Science
- PubMed
- Google Scholar
- Transport Research International Documentation (TRID)
- WHO Institutional Repository
- World Bank Open Knowledge Repository
- NRSA official publications and crash reports

Grey literature searching is recommended in governance and transport reviews to capture enforcement audits, crash statistics and policy diagnostics often absent from peer‐reviewed journals [17, 21].

Search strings combined behavioural, geographic and policy terms using Boolean operators:

- ‘reckless driving’ OR ‘dangerous driving’ OR ‘aggressive driving’ OR ‘speeding’ OR ‘traffic violations’
- AND ‘Ghana’ OR ‘sub‐Saharan Africa’
- AND ‘road traffic injuries’ OR ‘crashes’ OR ‘enforcement’ OR ‘policy reform’ OR ‘Safe Systems’ OR ‘economic cost’ OR ‘determinants’

The timeframe was limited to 2005–2025 to capture evidence from the United Nation (UN) Decade of Action for Road Safety (2011–2020) and SDG‐aligned reforms ([5, 22]. Foundational theoretical works were included selectively for conceptual grounding [2, 24]. English‐language publications were included due to feasibility constraints.

#### 3.3.1 Screening and Selection

Duplicate records were removed prior to screening. Titles and abstracts were independently screened by two reviewers to enhance reliability and reduce selection bias [23]. Full‐text articles were assessed against predefined eligibility criteria. Disagreements were resolved through discussion and consensus, consistent with PRISMA 2020 methodological guidance [20].

Institutional and governmental reports were retained where they demonstrated methodological transparency, statistical reliability and relevance to crash determinants, enforcement or policy evaluation [5, 6].

#### 3.3.2 Inclusion Criteria

Studies were eligible if they:

- Reported empirical findings on reckless, aggressive or dangerous driving in Ghana or SSA;
- Examined behavioural, socio‐economic, infrastructural or institutional determinants [2, 21];
- Assessed public health, economic or social consequences of road crashes [5];
- Evaluated enforcement reforms, technological interventions or Safe Systems strategies [12, 22];
- Were peer‐reviewed journal articles or reputable institutional publications.

#### 3.3.3 Exclusion Criteria

Studies were excluded if they:

- Focused exclusively on vehicle mechanics without behavioural or governance relevance;
- Addressed transport topics unrelated to road safety outcomes;
- Were opinion pieces lacking empirical grounding;
- Lacked sufficient methodological transparency in data reporting;
- Were published prior to 2005 unless conceptually foundational.

#### 3.3.4 PRISMA Flow Summary

The study selection process followed the PRISMA 2020 framework to ensure transparency and replicability [20]. Table 1 summarizes the identification, screening, eligibility and inclusion stages.

**TABLE 1:** Preferred Reporting Items for Systematic Reviews and Meta‐Analyses (PRISMA) flow of study selection.

PRISMA stage	Description of process	Outcome (*n*)
**Identification**	Records identified through database searching	1184
	Records identified through institutional repositories	142
	Additional records from reference screening	51
	Total records identified	1377
**Screening**	Duplicate records removed	236
	Records screened (titles and abstracts)	1141
	Records excluded at screening stage	892
**Eligibility**	Full‐text articles assessed for eligibility	249
	Full‐text articles excluded (outside scope, no empirical basis, unrelated focus, insufficient transparency, pre‐2005)	207
**Inclusion**	Studies included in qualitative synthesis	42
	Studies with quantitative crash/impact components	16

#### 3.3.5 Characteristics of Included Studies

Following the screening and eligibility assessment process, a total of 42 studies were included in the final qualitative synthesis. These studies comprised a diverse body of evidence encompassing quantitative, qualitative and mixed‐methods research designs, as well as methodologically transparent institutional and policy reports. The inclusion of multiple forms of evidence was considered necessary given the multidisciplinary nature of road safety research, which spans behavioural science, public health, transport engineering, economics and governance studies.

The selected studies varied in geographical focus, methodological approach and thematic emphasis. Although the majority examined road safety issues within Ghana, several studies provided broader SSA perspectives or international comparative evidence relevant to Safe Systems reforms and road safety governance. The studies collectively addressed key themes aligned with the objectives of the review, including the behavioural and structural determinants of reckless driving, the socio‐economic and public health consequences of road traffic crashes and the effectiveness of regulatory and institutional interventions designed to improve road safety outcomes. To provide a transparent overview of the evidence base underpinning this review, Table 2 summarizes the key characteristics of the included studies, including study design, geographical focus, thematic coverage, data sources and publication period. This summary demonstrates the breadth and diversity of the evidence synthesized and provides context for the narrative analysis presented in subsequent sections.

**TABLE 2:** Characteristics of studies included in the systematic review (*N* = 42).

Characteristic	Category	Number of studies (*n*)	Percentage
**Study design**	Quantitative	16	38.1
	Qualitative	18	42.9
	Mixed methods	8	19.0
**Geographical focus**	Ghana‐specific studies	24	57.1
	Sub‐Saharan African studies	10	23.8
	International comparative studies	8	19.1
**Primary research focus**	Determinants of reckless driving	15	35.7
	Consequences of road traffic crashes	10	23.8
	Enforcement and regulatory interventions	8	19.0
	Road safety governance and institutional capacity	5	11.9
	Safe Systems and comparative reform strategies	4	9.6
**Data source**	Peer‐reviewed journal articles	30	71.4
	Government and institutional reports	12	28.6
**Publication period**	2005–2010	7	16.7
	2011–2015	9	21.4
	2016–2020	12	28.6
	2021–2025	14	33.3

*Note:* Authors’ synthesis of included studies (2025).

The 42 studies included in the review represent a diverse evidence base encompassing quantitative, qualitative and mixed‐methods research designs (Table 2). Quantitative studies accounted for 38.1% of the included evidence and primarily focused on crash patterns, injury outcomes and enforcement effectiveness. Qualitative and mixed‐methods studies provided valuable insights into behavioural norms, institutional capacity, governance challenges and road safety policy implementation. More than half of the studies focused specifically on Ghana, whereas the remainder provided regional SSA evidence or international comparative perspectives relevant to Safe Systems reforms. The majority of sources were peer‐reviewed journal articles, supplemented by institutional reports from organizations such as NRSA, WHO and the Organisation for Economic Co‐operation and Development (OECD). Collectively, these studies provided a sufficiently broad evidence base to support a comprehensive narrative synthesis of the determinants, consequences and regulatory responses associated with reckless driving in Ghana.

### 3.4 Data Extraction and Analysis

Data were extracted using a standardized coding framework consistent with qualitative content analysis procedures [25]. Extracted variables included: country, study design, sample characteristics, identified determinants, crash outcomes, enforcement mechanisms, policy interventions and key findings.

Experimental and quasi‐experimental crash studies (*n* = 16) were prioritized when interpreting causal impacts, consistent with hierarchies of evidence in policy evaluation [23]. However, qualitative and mixed‐methods studies (*n* = 26) were essential for understanding institutional capacity, enforcement credibility, corruption risks, economic pressures on commercial drivers and governance constraints.

Given heterogeneity in outcome measures and methodological approaches, statistical meta‐analysis was not appropriate. Instead, a narrative synthesis approach was employed [26]. Narrative synthesis is particularly suitable where contextual variability and outcome diversity limit quantitative aggregation [21].

Findings were organized into three analytical domains aligned with the study objectives:

- Determinants of reckless driving (behavioural, socio‐economic, institutional and infrastructural);
- Public health, economic and social consequences;
- Comparative reform strategies and Safe Systems interventions adaptable to Ghana.

This structured narrative synthesis enhances interpretive coherence, supports cross‐context comparison and strengthens the review's policy relevance for sustainable road safety reform in Ghana.

## 4 Results

### 4.1 Multidimensional Determinants of Reckless Driving in Ghana

**FIGURE 2:**
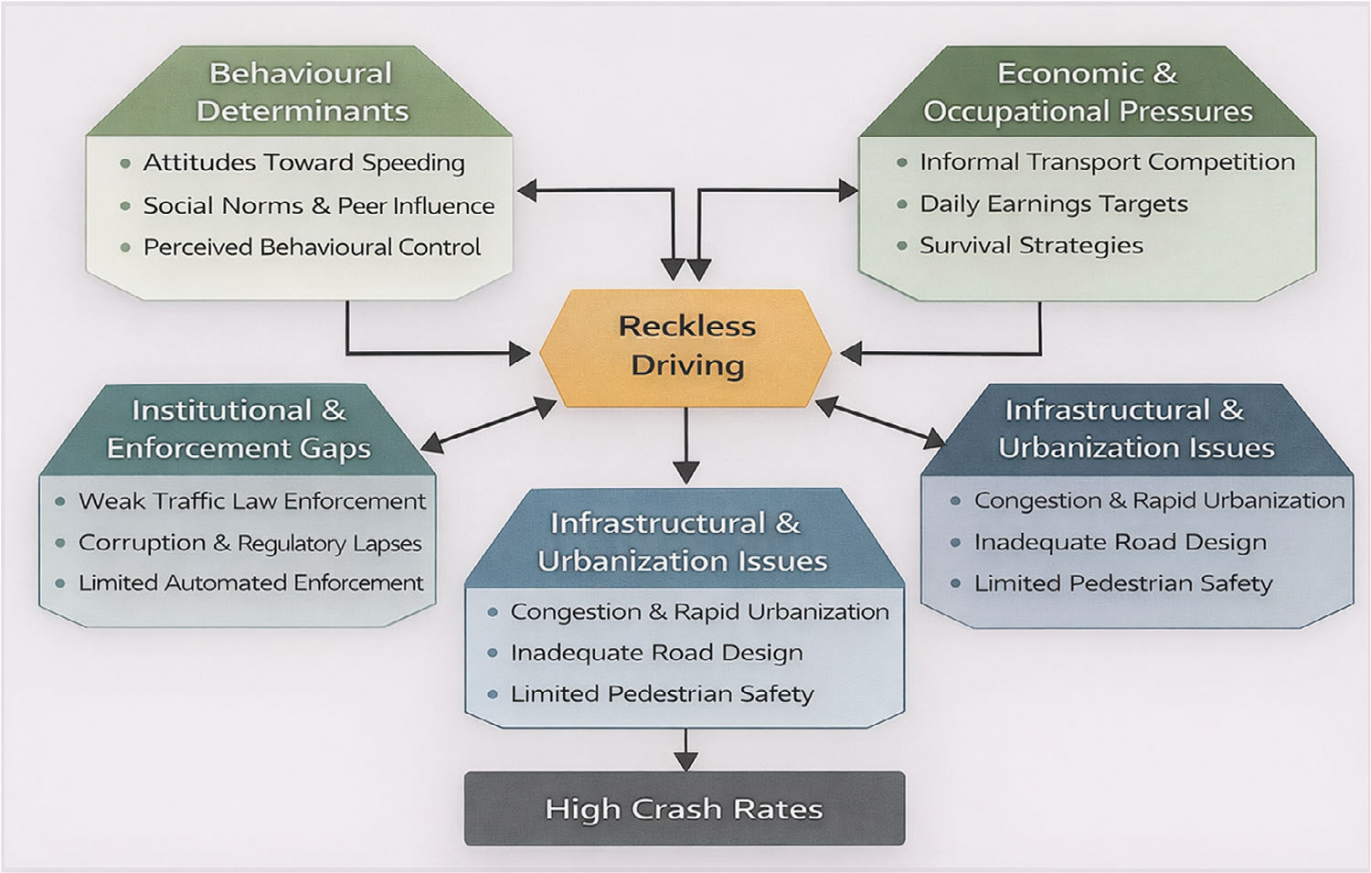
Multidimensional determinants of reckless driving in Ghana.

The synthesis of 42 included studies reveals that reckless driving in Ghana is shaped by interacting behavioural, structural, institutional and economic determinants. Across the reviewed empirical crash reports and enforcement audits, human factors account for approximately 85%–90% of recorded crashes, with speeding, dangerous overtaking, inattentiveness and lane indiscipline consistently identified as leading proximate causes [5, 6]. Within the integrated TPB–SSsA framework guiding this review, these determinants operate at two interconnected levels: behavioural drivers influencing driver decision‐making and systemic conditions shaping exposure to risk and crash severity (Figure 2).

#### 4.1.1 Behavioural Determinants

Speeding emerged as the most frequently cited traffic violation. NRSA reports indicate that speeding contributes to approximately 50%–60% of fatal crashes in Ghana, although these estimates vary considerably across reporting periods [6]. However, these figures should be interpreted with caution, as they are primarily derived from police and administrative crash records, which are known to suffer from significant underreporting when compared with hospital‐based data [27, 28, 29]. Despite these limitations, the available evidence consistently identifies speeding as one of the leading contributors to fatal road crashes in Ghana [6, 30]. Nevertheless, the absence of robust multi‐source data integration limits a more precise understanding of the relative contribution of speeding compared to other risk factors, such as drunk driving, poor road infrastructure and vehicle unfitness. This highlights the urgent need for improved road safety surveillance systems that triangulate police, hospital and insurance records to generate more reliable statistics for evidence‐based interventions. From the perspective of the TPB, these patterns reflect how driver intentions are shaped by permissive attitudes toward speeding, prevailing social norms within commercial transport networks and perceived behavioural control influenced by weak enforcement environments.

Drivers often perceive speeding as economically rational and socially normalized. Under competitive commercial transport conditions, particularly among trotro operators, time efficiency directly affects income. The perception that ‘everyone speeds’ reinforces subjective norms, whereas weak enforcement reduces perceived risk of punishment. These findings correspond directly with TPB constructs, where subjective norms and perceived behavioural control influence behavioural intentions and increase the likelihood of risk‐taking driving practices. Optimism bias, where drivers underestimate personal crash risk, further reinforces risk‐taking [4].

The reviewed literature also highlights specific transport corridors where speeding, dangerous overtaking and other risky driving behaviours are more frequently reported. Although the studies included in this review did not provide sufficient geo‐referenced data to support the development of a scientifically robust spatial map, the evidence nevertheless points to several major road corridors that are consistently associated with elevated road safety risks. Table 3 summarizes the major high‐risk corridors and the dominant risk factors identified across the reviewed studies.

**TABLE 3:** Major high‐risk road corridors and reported risk factors in Ghana.

Road corridor	Reported risk factors
Accra–Kumasi Highway	Speeding, dangerous overtaking, heavy commercial traffic
Accra–Cape Coast Highway	Excessive speed, pedestrian conflicts, weak speed enforcement
Accra–Tema Motorway	Speed violations, congestion‐related risk‐taking, lane indiscipline
Kumasi Metropolitan Corridors	Traffic congestion, abrupt lane switching, signal violations
Major intercity highways	Limited speed monitoring, enforcement gaps, commercial transport competition

As shown in Table 3, the Accra–Kumasi and Accra–Cape Coast corridors are repeatedly identified in the literature as locations where commercial transport pressures, speeding and overtaking behaviours elevate crash risks. Similarly, urban corridors within Accra and Kumasi experience high levels of congestion‐related violations, including abrupt lane switching and signal non‐compliance. These patterns suggest that reckless driving in Ghana is not evenly distributed across the road network but tends to concentrate along major commercial and high‐mobility corridors where traffic volumes, economic pressures and enforcement challenges intersect. This finding reinforces the need for targeted enforcement, speed management interventions and infrastructure improvements in identified high‐risk locations.

#### 4.1.2 Economic and Occupational Pressures

Approximately 70% of passenger transport in urban Ghana is provided by informal or semi‐formal commercial operators. Evidence suggests that daily target‐based earnings systems incentivize rapid passenger turnover, encouraging speeding and aggressive overtaking [10]. Economic precarity increases tolerance for rule violations, illustrating TPB's emphasis on perceived behavioural control: When drivers believe enforcement is inconsistent, risky driving becomes a rational survival strategy. In this context, economic incentives interact with behavioural motivations, demonstrating how external pressures reinforce driver intentions to engage in unsafe practices.

#### 4.1.3 Institutional and Enforcement Gaps

Institutional weaknesses compound behavioural risks. Ghana's traffic law enforcement capacity remains limited relative to vehicle growth. Vehicle registrations have increased steadily over the past decade, whereas police‐to‐vehicle ratios remain low. Automated speed enforcement and red‐light cameras remain limited in coverage. Available reports suggest that systematic speed‐monitoring coverage remains limited and may cover less than one‐fifth of major highway networks [6].

From the perspective of SSsA, these enforcement gaps represent systemic weaknesses that reduce the capacity of the transport system to mitigate human error. Under SSsA principles, road safety is not dependent solely on individual compliance but on the ability of institutions, infrastructure and enforcement mechanisms to prevent errors from resulting in severe injuries or fatalities [12]. In Ghana, infrastructural deficits, poor road markings, inadequate pedestrian crossings and limited traffic calming reduce system resilience. For example, pedestrian fatalities account for roughly 35%–40% of total road deaths, indicating design vulnerabilities [5]. This pattern reflects a central premise of SSsA that unsafe system design can amplify the consequences of inevitable human mistakes.

#### 4.1.4 Infrastructure and Urbanization

Rapid urbanization in Accra and Kumasi has outpaced road expansion. Traffic congestion contributes indirectly to reckless behaviours such as abrupt lane switching and signal violations. Empirical modelling studies indicate that high‐density corridors experience disproportionately higher crash rates per vehicle kilometre travelled [31]. Within the SSsA framework, these findings highlight how infrastructural limitations and rapid urban growth increase systemic exposure to risk, reinforcing the need for safer road design and traffic management interventions.

#### 4.1.5 Licensing and Driver Training

Licensing irregularities and inconsistent driver training standards also contribute to unsafe driving practices. Studies indicate that knowledge of traffic regulations among commercial drivers varies widely, with some surveys reporting that up to 30% of drivers demonstrate incomplete understanding of speed regulations and right‐of‐way rules. Weak monitoring of driving schools further undermines compliance. From a TPB perspective, insufficient training affects drivers’ perceived behavioural control and risk perception, whereas from an SSsA perspective, weak licensing oversight represents a systemic governance failure within the broader road safety system.

Overall, the evidence indicates that reckless driving in Ghana is sustained by feedback loops between economic incentives, weak deterrence, social normalization of violations and infrastructural limitations. The integration of TPB and the SSsA clarifies how behavioural motivations and systemic conditions interact to produce high crash rates. Although TPB explains why drivers engage in risk‐taking behaviours, SSsA highlights how institutional weaknesses and infrastructural deficits fail to prevent those behaviours from translating into severe RTIs and fatalities. The determinants of reckless driving in Ghana are, therefore, multidimensional and mutually reinforcing.

### 4.2 Socio‐Economic and Health Consequences of Reckless Driving in Ghana

**FIGURE 3:**
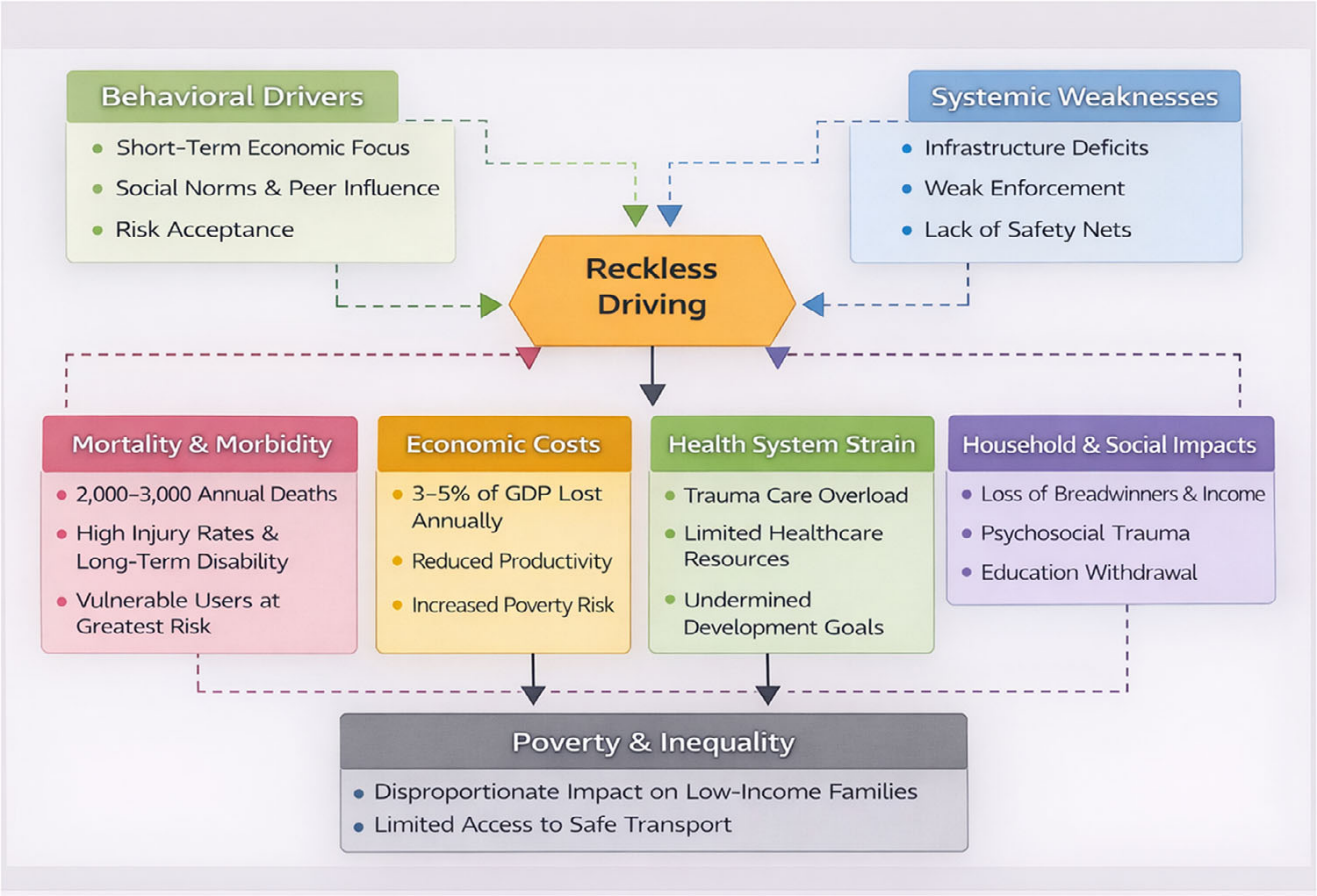
Socio‐economic and health consequences of reckless driving in Ghana. GDP, gross domestic product.

The reviewed evidence demonstrates that reckless driving in Ghana generates profound public health, economic and social consequences that extend beyond individual crash victims (Figure 3). RTIs remain one of the leading causes of premature mortality globally, with approximately 1.19 million deaths annually [1]. SSA records the highest regional fatality rate per 100,000 population, and Ghana's burden remains substantial relative to its vehicle fleet size [13, 14]. Within the integrated TPB–SSsA framework guiding this review, these consequences reflect the combined effects of individual behavioural decisions and systemic safety deficiencies within the road transport system.

#### 4.2.1 Mortality and Injury Burden

National statistics indicate that Ghana records between 2000 and 3000 road traffic deaths annually, with tens of thousands sustaining varying degrees of injury [6]. Young adults aged 18–35 constitute the majority of victims, reflecting the demographic most engaged in commercial driving and economically productive activities.

Pedestrians account for approximately 35%–40% of fatalities, motorcyclists about 20%–25%, and vehicle occupants the remainder [5]. These figures highlight systemic vulnerabilities in road design and speed management. From the perspective of SSsA, such patterns indicate that the transport system lacks adequate protective mechanisms capable of accommodating human error while safeguarding vulnerable road users.

RTIs made up 32% of trauma admissions (range 4%–91%) in 83 hospital‐based studies in SSA [32]. A research at Komfo Anokye Teaching Hospital in Kumasi indicated that 49.6% of emergency department patients had vehicular‐related trauma, with 26.1% having brain injuries [33]. Road injuries predominated among trauma cases [34]. Road traffic accidents caused 71.9% of traumatic brain injuries in Cape Coast, Ghana showed [35]. Long‐term disability, including limb loss, spinal cord injury and traumatic brain injury, contributes significantly to the burden of disease. Within the SSsA framework, these outcomes demonstrate how high‐speed collisions and unsafe road environments amplify the severity of crashes once behavioural errors occur.

#### 4.2.2 Economic Costs

The economic implications of reckless driving are substantial. WHO estimates that road traffic crashes cost countries between 3% and 5% of gross domestic product (GDP) annually [5]. Applying conservative estimates to Ghana suggests annual economic losses amounting to billions of Ghana cedis through healthcare expenditure, lost productivity, vehicle damage, insurance claims and infrastructure repair.

Productivity losses are particularly severe because the majority of victims fall within the working‐age population. Empirical economic modelling in SSA indicates that each fatal crash represents not only immediate medical costs but also long‐term household income loss, reduced educational attainment for dependents and increased poverty risk [21].

From a TPB perspective, these macroeconomic consequences emerge from cumulative individual behavioural decisions. Drivers may prioritize immediate financial gains, such as faster passenger turnover, while underestimating long‐term societal costs associated with unsafe driving behaviours.

However, the aggregate cost to the national economy far outweighs these short‐term benefits. The misalignment between individual incentives and collective welfare highlights the systemic governance challenges emphasized by SSsA, where institutional interventions are necessary to correct behavioural risk patterns.

#### 4.2.3 Health System Strain

Reckless driving places significant strain on Ghana's health system. Emergency departments in urban centres report frequent surges in trauma cases following weekend and holiday travel peaks. Road crashes demand high‐cost interventions, including surgical procedures, intensive care, imaging and long‐term rehabilitation.

Trauma care expenditure diverts limited healthcare resources from preventive and primary care services. In lower middle‐income contexts, where health financing is already constrained, such diversion undermines broader development goals. Within the Safe Systems framework, this pattern illustrates how failures in upstream road safety governance generate downstream burdens for healthcare systems.

#### 4.2.4 Household and Social Consequences

At the household level, road fatalities often eliminate primary breadwinners. Qualitative evidence indicates that surviving family members frequently experience financial instability, withdrawal of children from school and long‐term indebtedness. Women and children disproportionately bear secondary economic consequences.

Psychological trauma also emerges as a significant yet underreported outcome. Survivors may experience post‐traumatic stress disorder (PTSD), anxiety, and depression. Families of deceased victims often face prolonged grief and social dislocation. These impacts illustrate the broader social consequences of behavioural risk patterns highlighted in TPB, where individual driving decisions can generate cascading social effects beyond the immediate crash event.

#### 4.2.5 Poverty and Inequality Dimensions

RTIs have regressive effects, disproportionately affecting lower income populations who rely on public and informal transport. Pedestrians and roadside traders, often from economically vulnerable groups, face elevated exposure risks. Studies show that vulnerable road users account for more than half of fatalities in many SSA countries [5].

From a systems perspective, inequitable infrastructure, such as lack of sidewalks, pedestrian bridges and traffic calming in low‐income neighbourhoods, reflects distributional injustice. SSsA emphasizes that road networks must be designed to protect all users, particularly vulnerable populations who face the highest exposure to traffic risks.

#### 4.2.6 Macroeconomic and Developmental Implications

Reckless driving undermines Ghana's progress toward SDG 3.6, which aims to halve global road traffic deaths and injuries. Persistent crash rates weaken investor confidence, strain insurance markets and impose fiscal burdens on public institutions.

Economic growth depends on mobility efficiency and workforce stability. When road systems generate high fatality rates, the developmental dividend of urbanization is compromised. Evidence suggests that countries achieving sustained reductions in traffic fatalities, such as Sweden under Vision Zero, experience long‐term economic and social gains [12, 22].

Within the integrated TPB–SSsA framework, these macro‐level consequences reflect the interaction between behavioural risk‐taking and systemic safety deficiencies. Although TPB explains how individual driver decisions contribute to crash occurrence, SSsA highlights how weaknesses in infrastructure design, enforcement systems and institutional governance amplify the societal impact of those behaviours.

In summary, the socio‐economic and health consequences of reckless driving in Ghana are multidimensional and severe. The evidence indicates that behavioural motivations and systemic safety gaps interact to produce widespread public health, economic and social costs across multiple levels of society.

### 4.3 Global Best Practices and Evidence‐Based Recommendations for Sustainable Road Safety Reform

**FIGURE 4:**
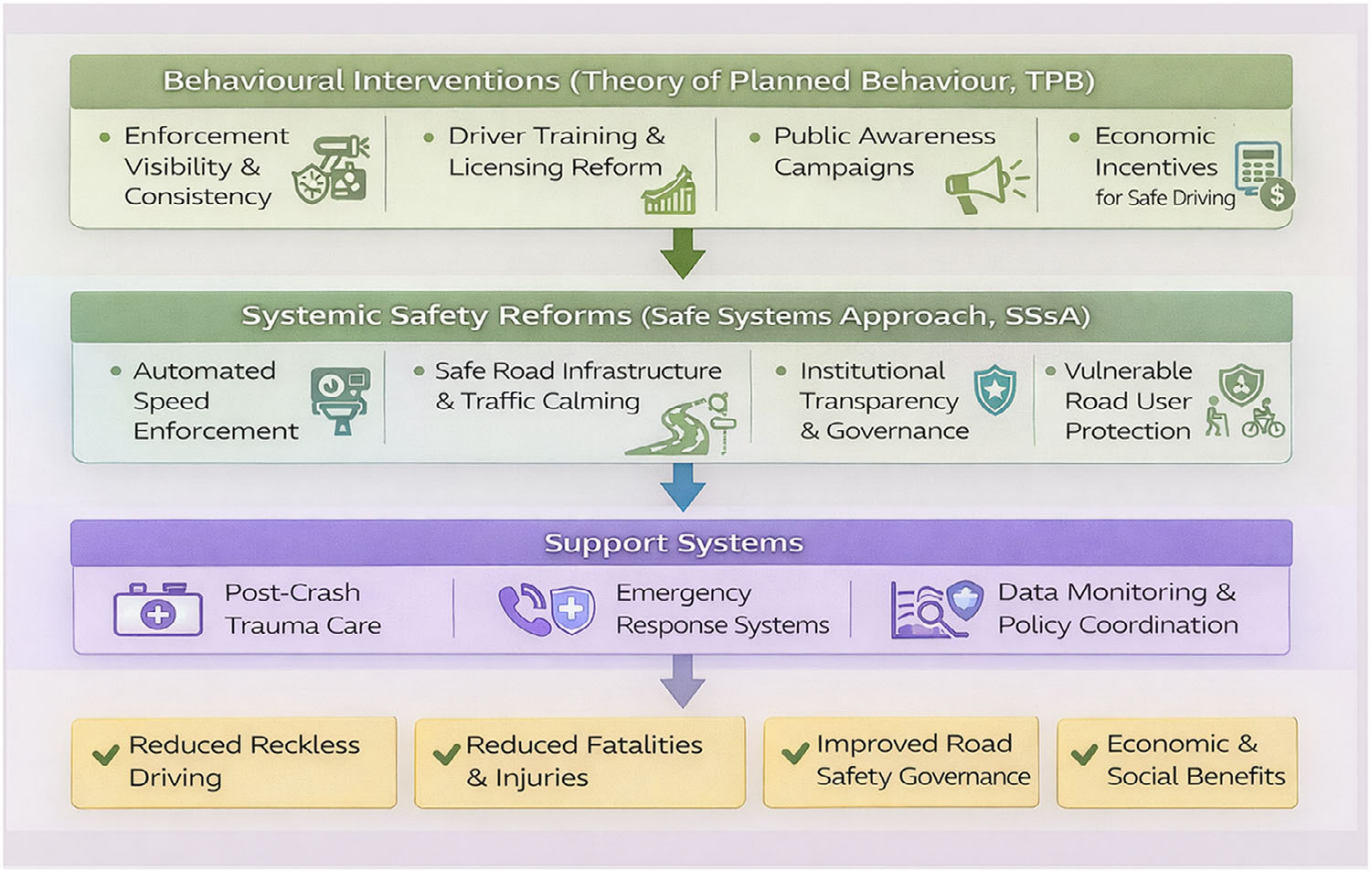
Integrated global best practices framework for reducing reckless driving and road traffic fatalities in Ghana.

The comparative synthesis of 42 studies demonstrates that sustained reductions in reckless driving and road traffic fatalities are achievable when behavioural interventions are integrated within systemic institutional reforms. Drawing on the SSsA and the TPB, this section identifies evidence‐based global practices adaptable to Ghana as presented in Figure 4.

#### 4.3.1 Speed Management and Automated Enforcement

Speeding accounts for approximately 50%–60% of fatal crashes in Ghana [6].

International evidence shows that automated speed enforcement significantly reduces both average speeds and fatality rates. For example, widespread speed camera deployment in Australia led to reductions of 20%–30% in fatal crashes in monitored corridors [22]. Similarly, under Vision Zero reforms in Sweden, systematic speed control and road redesign contributed to one of the world's lowest road fatality rates, approximately 2–3 deaths per 100,000 population compared to the global average of over 15 per 100,000 [5].

Under the Safe Systems framework, speed management is foundational because human tolerance to crash forces is biologically limited. Infrastructure‐based measures such as speed humps, roundabouts and median barriers can reduce severe injury risk by up to 40%–50% [21]. In Ghana, scaling automated speed cameras and expanding traffic calming in high‐risk corridors could substantially reduce fatality exposure.

From a TPB perspective, consistent enforcement alters subjective norms and perceived behavioural control. When drivers believe speeding will reliably result in sanctions, compliance increases. Evidence indicates that visible enforcement combined with penalty points systems significantly shifts driver attitudes toward risk.

#### 4.3.2 Graduated Licensing and Driver Training Reform

Weak driver training and licensing irregularities contribute to risky behaviour in Ghana. Countries such as Canada and Australia have implemented graduated driver licensing (GDL) systems that restrict high‐risk driving conditions for novice drivers. Studies show that GDL systems reduce crash risk among young drivers by 20%–40% [22].

Incorporating mandatory hazard perception training, periodic re‐certification for commercial drivers and digitalized licensing databases could address institutional weaknesses. Under TPB, enhanced training influences attitudes toward safety and strengthens perceived control over safe driving behaviours.

#### 4.3.3 Safe Infrastructure and Vulnerable Road User Protection

Pedestrians account for roughly 35%–40% of road fatalities in Ghana [5]. Countries adopting Safe Systems engineering, such as Sweden, prioritize separation of pedestrians from high‐speed traffic. Roundabout conversion alone has been associated with up to 70% reductions in severe intersection crashes [21].

In Ghanaian urban centres like Accra and Kumasi, expanding pedestrian bridges, sidewalks, protected crossings and dedicated motorcycle lanes could significantly reduce exposure risk. The Safe Systems principle emphasizes designing roads that accommodate inevitable human error rather than assuming perfect compliance.

#### 4.3.4 Anti‐Corruption and Institutional Strengthening

Enforcement credibility remains a challenge in many SSA contexts. Evidence suggests that perceived corruption reduces deterrence effectiveness and weakens subjective norms regarding compliance. Digital ticketing systems, transparent penalty point records and independent oversight mechanisms have been effective in strengthening accountability in countries such as Singapore.

Institutional reform aligns directly with the Safe Systems philosophy, which distributes responsibility across governance structures rather than individual drivers alone. Strong institutions reduce behavioural rationalizations that undermine compliance.

#### 4.3.5 Public Awareness and Behavioural Change Campaigns

Public education campaigns are most effective when combined with visible enforcement. Meta‐analyses indicate that awareness campaigns alone yield modest reductions (5%–10%) in violations, but when paired with enforcement, reductions can exceed 20% [21].

Under TPB, campaigns reshape attitudes and social norms. In Ghana, targeted messaging for commercial drivers, emphasizing economic losses from crashes rather than solely moral appeals, may be more effective in shifting behaviour.

#### 4.3.6 Economic Incentives and Insurance Reform

Usage‐based insurance schemes and fleet safety audits have reduced crash rates in parts of Europe and North America. Incentivizing safe driving through premium discounts or tax rebates aligns economic incentives with safety outcomes.

In Ghana's commercial transport sector, linking licensing renewal to documented safety compliance and crash‐free records could alter cost–benefit calculations influencing driver behaviour.

#### 4.3.7 Post‐Crash Care and Trauma Systems

Although prevention remains the cornerstone of road safety reform, strengthening post‐crash care is critical for reducing fatality severity and long‐term disability. Evidence indicates that rapid and well‐coordinated emergency response systems can reduce post‐crash mortality by up to 25% [5]. In the Ghanaian context, expanding ambulance coverage, improving dispatch coordination and enhancing trauma training for first responders would significantly improve survivability outcomes. Strengthening referral systems between district facilities and tertiary trauma centres is equally important to ensure timely and effective treatment. Although post‐crash care does not prevent crashes, it mitigates their human and economic consequences, reinforcing the broader Safe Systems commitment to shared responsibility across the transport and health sectors.

Synthesizing global evidence suggests that sustainable road safety reform in Ghana requires a coordinated and multidimensional strategy. Central to this model is the expansion of automated and visible speed enforcement to recalibrate deterrence and reshape compliance norms. This must be complemented by infrastructure redesign that prioritizes vulnerable road users, including pedestrians and motorcyclists, through traffic calming, protected crossings and safer intersection design. Modernizing licensing systems and strengthening driver training standards are essential to improving professional competence and accountability, particularly within the commercial transport sector. Institutional transparency and anti‐corruption safeguards are equally necessary to restore enforcement credibility and public trust. Aligning economic incentives with safety outcomes, through insurance reforms and commercial transport regulation, can further shift the cost–benefit calculations that often underpin risk‐taking behaviour. Finally, strengthening trauma care systems ensures that when crashes do occur, survivability and recovery outcomes are maximized.

Under the SSsA, these reforms function collectively to reduce systemic risk rather than operating as isolated interventions. The TPB complements this framework by addressing the motivational and normative factors that shape driver conduct. Quantitative evidence from countries implementing comprehensive Safe Systems reforms demonstrates sustained fatality reductions exceeding 30%–50% over time [22]. If Ghana achieved even a conservative 30% reduction from current annual fatalities, approximately 2500 deaths, this would translate into roughly 750 lives saved each year, alongside significant reductions in healthcare costs and productivity losses. Such gains would yield substantial social and economic returns while advancing national development objectives.

#### 4.3.8 Critical Evaluation of Ghana's Regulatory Responses

Ghana has implemented several regulatory interventions to improve road safety, including the Road Traffic Act, 2004 (Act 683), enforcement activities by the Motor Traffic and Transport Department (MTTD), public awareness campaigns coordinated by NRSA, licensing reforms and periodic speed enforcement exercises. These interventions reflect a sustained policy commitment to reducing road traffic crashes and fatalities. Nevertheless, the persistence of high crash rates and recurring traffic violations suggests that implementation challenges continue to undermine their overall effectiveness [6, 11]. This indicates that the existence of regulatory frameworks alone is insufficient without corresponding institutional capacity and enforcement effectiveness.

The Road Traffic Act, 2004, (Act 683) provides a comprehensive legal framework governing driver behaviour, vehicle standards, licensing requirements and traffic management practices [36]. However, the effectiveness of the legislation has been constrained by enforcement gaps. Evidence suggests that traffic law enforcement remains inconsistent, particularly outside major urban centres, where limited personnel, inadequate logistical resources, insufficient monitoring technologies and perceptions of corruption weaken deterrence [7, 11]. Consequently, compliance often depends less on the existence of legal sanctions and more on drivers’ perceptions of the likelihood of detection and punishment.

The NRSA has also implemented sustained road safety education campaigns, media sensitization programmes and stakeholder engagement initiatives aimed at promoting safer road‐user behaviour. These interventions have contributed to increased public awareness of road safety risks and traffic regulations [6]. However, the evidence reviewed suggests that awareness alone has not translated into sustained behavioural change. The continued prevalence of speeding, dangerous overtaking, distracted driving and other traffic violations indicates a persistent gap between knowledge and compliance [10, 11, 27]. This finding highlights the limitations of educational approaches when implemented without complementary enforcement and institutional accountability mechanisms.

Similarly, MTTD enforcement operations have contributed to periodic reductions in traffic violations, particularly during festive seasons and targeted road safety campaigns. However, these enforcement efforts are often episodic and reactive rather than continuous and preventive in nature. As a result, their long‐term deterrent effect may be limited. Comparative evidence from countries that have successfully reduced road traffic fatalities demonstrates that sustained improvements are more likely when traditional policing is complemented by automated enforcement technologies, including speed cameras, red‐light cameras and digital monitoring systems [22]. In comparison, Ghana's enforcement infrastructure remains relatively underdeveloped, limiting its ability to achieve consistent compliance across the road network [6].

Licensing reforms and efforts to strengthen driver training have improved regulatory oversight and enhanced professional standards within parts of the transport sector. Nevertheless, concerns remain regarding variability in the quality of driver training, weak monitoring of driving schools and inconsistencies in the assessment of driver competence [10, 11]. These weaknesses reduce the effectiveness of licensing systems as instruments for promoting safe driving behaviour and ensuring that drivers possess the requisite skills and knowledge to operate vehicles safely.

The literature also suggests that Ghana's road safety governance framework faces broader institutional challenges relating to coordination, monitoring and accountability. Although multiple agencies share responsibility for road safety management, fragmented implementation and resource constraints often hinder effective collaboration [11]. Furthermore, weaknesses in road crash data collection and integration continue to limit evidence‐based policymaking. Studies have identified discrepancies between police‐recorded crash statistics and hospital‐based injury surveillance systems, raising concerns about underreporting and the reliability of available data for planning and evaluation [27]. These challenges make it difficult to accurately assess the effectiveness of existing interventions and to identify priority areas for reform.

Overall, the evidence suggests that Ghana's regulatory framework is not fundamentally deficient. Rather, the principal challenges lie in implementation capacity, enforcement consistency, technological modernization, institutional coordination, data quality and accountability mechanisms [1, 11, 22]. Therefore, future reforms should focus not only on introducing new regulations but also on strengthening the institutions responsible for implementing, monitoring and enforcing existing road safety policies. Such an approach is consistent with the SSsA, which emphasizes that sustainable reductions in road traffic fatalities require strong institutions, effective enforcement systems, safer infrastructure, reliable data systems and shared responsibility among all stakeholders within the transport sector.

## 5 Discussion

This review synthesized multidisciplinary evidence to examine the determinants, consequences and reform pathways related to reckless driving in Ghana. The findings confirm that reckless driving is not merely a matter of individual misconduct but a systemic governance and public health challenge shaped by behavioural norms, economic incentives, infrastructural design and institutional capacity. By integrating the TPB and the SSsA, this discussion situates Ghana's road safety crisis within broader global reform trajectories while offering a theoretically grounded interpretation of the results.

The evidence shows that speeding contributes to approximately 50%–60% of fatal crashes in Ghana [6], whereas pedestrians account for nearly 35%–40% of total fatalities [5]. These figures highlight the interaction between high‐risk driving behaviour and infrastructural vulnerability. Excessive speed increases both crash likelihood and injury severity, particularly in environments lacking protective pedestrian infrastructure, traffic calming and effective enforcement.

However, these statistics should be interpreted with caution. Much of the available road safety evidence in Ghana is derived from police and NRSA records, which may not fully capture the true burden of RTIs and fatalities. Evidence from low‐ and middle‐income countries has consistently shown discrepancies between police‐reported crash data and hospital‐based injury surveillance systems, suggesting that fatalities and serious injuries are often underreported. Furthermore, the attribution of crashes to specific causes, such as speeding, frequently relies on post‐crash investigations and administrative assessments that may be affected by reporting limitations, incomplete information or inconsistencies in investigative procedures. Consequently, the reported proportions should be regarded as indicative rather than definitive estimates.

Notwithstanding these limitations, the broader pattern of evidence remains consistent across studies and data sources. Speeding, inadequate protection of vulnerable road users, weak enforcement and infrastructural deficiencies repeatedly emerge as major contributors to road traffic fatalities. The convergence of findings across institutional reports and empirical studies therefore provides reasonable confidence in the overall conclusions of this review, even though the precise magnitude of individual risk factors may vary.

From a TPB perspective [24], reckless driving persists because attitudes toward speeding are often permissive, subjective norms within commercial driving communities normalize rule violations, and perceived behavioural control is shaped by weak and inconsistent enforcement. Commercial drivers frequently weigh immediate financial gains, such as increased passenger turnover, against a low perceived probability of sanction. When enforcement is sporadic or perceived as negotiable, deterrence weakens, reinforcing a cycle of calculated risk‐taking. In this context, risky driving becomes economically rational at the individual level, even though it produces substantial collective harm.

However, attributing crashes solely to individual decision‐making is analytically insufficient. The SSsA [12] reframes reckless driving as a manifestation of systemic failure rather than isolated human error. In Ghana, limited automated speed enforcement coverage, estimated at below 20% of major highways, combined with inadequate pedestrian infrastructure, insufficient traffic calming and inconsistent licensing oversight reduces systemic resilience. Where road environments permit high‐speed interaction between vehicles and vulnerable users, inevitable human error becomes fatal. Thus, behavioural violations and infrastructural weaknesses operate as mutually reinforcing feedback loops: Economic pressure encourages speeding; weak enforcement lowers perceived costs; infrastructural deficits amplify crash severity; and institutional constraints limit corrective capacity.

The evidence base also reveals important methodological and interpretive challenges. Although some studies identify behavioural factors, particularly speeding, dangerous overtaking and driver inattentiveness, as the dominant determinants of road crashes, others place greater emphasis on infrastructural deficits, institutional weaknesses and enforcement failures. These variations may reflect differences in study design, geographical focus, measurement approaches and data sources. Rather than representing contradictory findings, they suggest that reckless driving should be understood as a multidimensional phenomenon shaped by the interaction of behavioural, environmental and governance‐related factors. This interpretation is consistent with both TPB and SSsA, which emphasize the interconnected nature of individual behaviour and systemic conditions.

The socio‐economic implications elevate reckless driving from a transport concern to a development crisis. Ghana records between 2000 and 3000 road traffic fatalities annually, with national economic losses estimated at 3%–5% of GDP [5]. Because victims disproportionately fall within the economically active age group, productivity losses extend beyond immediate mortality, affecting household income stability, educational attainment of dependents and long‐term poverty dynamics. Trauma admissions, accounting for up to 30% of emergency cases in major hospitals, place additional strain on already resource‐constrained health systems. Reducing crash incidence would therefore generate not only humanitarian benefits but also fiscal and macroeconomic gains.

These findings align with global evidence that RTIs disproportionately affect low‐ and middle‐income countries and vulnerable road users. The concentration of pedestrian fatalities in Ghana underscores structural inequities in transport planning. The SSsA emphasizes equitable protection across all road users; therefore, inadequate pedestrian safeguards reflect systemic design shortcomings rather than mere behavioural deviance. Failure to protect vulnerable road users represents both a safety deficit and a social justice concern.

Comparative evidence from Sweden and Australia demonstrates that sustained fatality reductions of 30%–50% are achievable through integrated Safe Systems strategies [22]. Sweden's fatality rate of approximately 2–3 per 100,000 population contrasts sharply with higher rates across SSA, underscoring the transformative potential of coordinated speed management, infrastructure redesign, enforcement modernization and institutional accountability. Crucially, these reductions were achieved not through behavioural messaging alone but through systemic redesign that anticipates human fallibility and limits crash severity.

Nevertheless, direct transplantation of high‐income country models without contextual adaptation would be inappropriate. Ghana's informal commercial transport sector, fiscal constraints and enforcement capacity limitations necessitate phased and scalable reform. Expanding automated speed enforcement in high‐risk corridors may yield immediate reductions, whereas comprehensive infrastructure redesign requires sustained capital investment and long‐term political commitment. Reform sequencing is therefore critical.

Under TPB, sustainable progress also requires normative change. Enforcement technologies are insufficient unless consistently applied and publicly visible. Behavioural change initiatives targeting commercial drivers should emphasize tangible economic consequences, vehicle repair costs, lost income, insurance penalties and legal sanctions, thereby aligning compliance with rational self‐interest rather than moral appeals alone. When compliance becomes economically advantageous and socially expected, subjective norms shift in favour of safety.

Institutional strengthening is central to recalibrating perceived behavioural control. Digitalized licensing systems, transparent penalty point databases, corruption‐resistant enforcement mechanisms and independent oversight can enhance enforcement credibility. When sanctions become predictable and unavoidable, compliance increases. Simultaneously, prioritizing vulnerable road user protection through sidewalks, protected crossings, traffic calming in residential areas, safer intersection design and speed‐sensitive urban planning addresses structural exposure risk and reduces crash severity.

**FIGURE 5:**
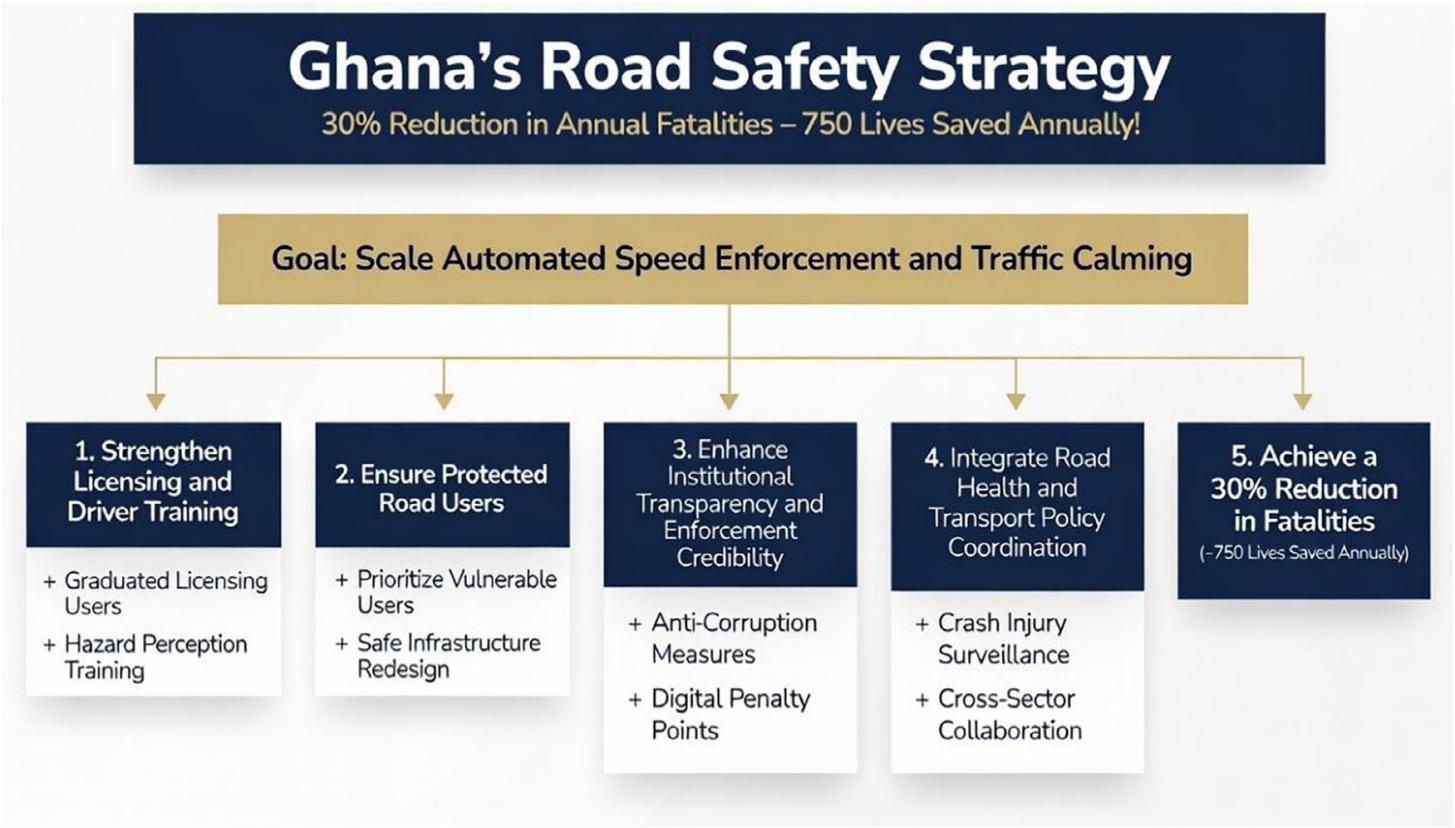
Integrated Safe Systems reform framework for achieving a 30% reduction in road traffic fatalities in Ghana.

The reform priorities identified in this review are synthesized in Figure 5, which presents an integrated safe systems reform framework for Ghana. The figure illustrates how behavioural interventions (licensing reform, driver training and enforcement credibility) and structural interventions (speed management, infrastructure redesign and public health coordination) operate as interconnected pillars rather than isolated strategies. By visually integrating these components, Figure 5 reinforces the central argument of this article: achieving a 30% reduction in annual fatalities, equivalent to approximately 750 lives saved each year, requires coordinated, multi‐sectoral action grounded in both TPB and SSsA. The framework, therefore, shifts the policy narrative from reactive crash response toward proactive system transformation.

The integration of TPB and SSsA constitutes an important theoretical contribution. TPB clarifies micro‐level motivational drivers of reckless behaviour, whereas SSsA elucidates macro‐level institutional and infrastructural determinants. Together, they demonstrate that behavioural reform and system redesign are complementary and mutually reinforcing. Addressing only individual attitudes without correcting structural risk exposure is unlikely to yield sustained impact; conversely, infrastructure improvements without behavioural accountability may limit gains.

If Ghana achieved a conservative 30% reduction in annual fatalities, approximately 750 lives would be saved each year, yielding substantial social and economic returns. Realizing this outcome requires scaling automated speed enforcement and traffic calming measures, strengthening licensing and driver training systems, ensuring institutional transparency and enforcement credibility, redesigning infrastructure to prioritize vulnerable road users and promoting integrated public health and transport policy coordination.

Ultimately, reckless driving in Ghana represents a preventable public health crisis. Transitioning from reactive crash response to proactive system redesign is not optional; it is imperative for sustainable development, economic resilience and the protection of human life.

### 5.1 Implications for Policy and Practice

The findings of this review underscore that reckless driving in Ghana is not merely a behavioural issue but a systemic governance and development challenge requiring coordinated institutional reform. For policymakers, the evidence points to the necessity of moving beyond isolated enforcement campaigns toward a structured Safe Systems strategy. Speed management should be prioritized through automated enforcement technologies, transparent penalty systems and consistent sanctioning. Enforcement credibility must be strengthened through digitalization and anti‐corruption safeguards to restore public trust and deterrence effectiveness.

Infrastructure redesign is equally critical. Urban planning authorities must integrate road safety into transport and land‐use policies by prioritizing pedestrian infrastructure, traffic calming, protected crossings and safer intersection designs. Vulnerable road users, particularly pedestrians and motorcyclists, should be central to infrastructure investment decisions. Designing forgiving road environments will significantly reduce the severity of inevitable human error.

For transport regulators, reform of commercial driver incentive systems is essential. Linking licensing renewal and insurance premiums to safety performance records would align economic incentives with compliance. Mandatory periodic re‐certification and hazard perception training for commercial drivers can improve professional standards. In parallel, structured engagement with transport unions can promote peer accountability and norm change within driver communities.

From a public health perspective, integrating trauma response strengthening into national road safety planning is vital. Improved emergency medical response systems and crash injury surveillance can reduce preventable mortality and enhance data‐driven policymaking.

For practitioners, including law enforcement agencies and civil society organizations, sustained public education campaigns should be paired with visible enforcement to reshape social norms around speeding and traffic violations. Behavioural change initiatives must address economic rationalizations for risk‐taking, emphasizing the long‐term financial and social costs of crashes.

Ultimately, sustainable road safety reform in Ghana requires cross‐sectoral coordination among transport authorities, police services, health institutions, insurance regulators and urban planners. Clear national targets aligned with global road safety goals, combined with measurable performance indicators, will be critical to ensuring accountability and sustained progress.

## 6 Limitations and Strengths

This review has several limitations. First, although it followed systematic review principles, it relied on available published and institutional reports, which may be subject to reporting bias and variations in methodological quality. Crash statistics in Ghana and parts of SSA may be underreported due to inconsistencies in data collection and reporting systems.

Second, the review did not undertake a formal risk‐of‐bias assessment or evidence grading exercise such as GRADE. Although efforts were made to prioritize methodologically robust studies and reputable institutional reports, variations in study quality may influence the strength of some conclusions. Future reviews should incorporate formal quality appraisal frameworks to enhance methodological rigour and strengthen confidence in synthesized findings.

Third, the heterogeneity of study designs and outcome measures limited the feasibility of conducting a formal meta‐analysis, necessitating reliance on narrative synthesis. Fourth, language restrictions to English‐language publications may have excluded relevant Francophone or Lusophone African studies. Finally, comparative evidence from high‐income countries may not fully capture contextual differences in institutional capacity, fiscal resources and informal transport structures.

Despite these limitations, the review demonstrates notable strengths. It integrates multidisciplinary evidence across public health, transport governance, behavioural psychology and economic analysis within a coherent analytical framework. The combined application of the TPB and the SSsA provides theoretical depth and conceptual clarity. The study moves beyond descriptive crash statistics to synthesize structural, behavioural and institutional determinants in an integrated manner. Additionally, the inclusion of quantitative evidence strengthens policy relevance, whereas the comparative orientation offers adaptable lessons for reform. By bridging global best practices with Ghana‐specific realities, the review contributes both scholarly insight and practical guidance for sustainable road safety transformation.

## 7 Conclusion

Reckless driving in Ghana represents a preventable public health crisis with significant socio‐economic consequences. The evidence synthesized in this review demonstrates that speeding, aggressive overtaking, weak enforcement, economic pressures on commercial drivers and infrastructural deficits collectively sustain high crash rates. Annual fatalities numbering in the thousands, alongside substantial injury burdens and economic losses estimated at several percentage points of national GDP, highlight the urgency of systemic reform.

The integration of the TPB and the SSsA reveals that reckless driving cannot be addressed through isolated behavioural campaigns alone. Individual risk‐taking behaviours are shaped by social norms, economic incentives and perceptions of enforcement credibility. At the same time, systemic weaknesses in infrastructure design, institutional coordination and regulatory oversight amplify the severity of inevitable human error.

Global evidence demonstrates that sustained fatality reductions of 30%–50% are achievable through coordinated enforcement, infrastructure redesign, licensing reform and institutional transparency. For Ghana, even modest reductions would translate into hundreds of lives saved annually, reduced healthcare costs and enhanced productivity.

Sustainable road safety reform requires long‐term political commitment, cross‐sectoral collaboration and data‐driven accountability. By shifting from reactive crash response to proactive system redesign, Ghana can align road safety governance with broader development objectives and safeguard the wellbeing of its citizens. Reckless driving is not an inevitable feature of mobility; it is a solvable challenge when evidence is translated into sustained action.

## Author Contributions

**Maame Adwoa Nyame Sam**: writing – original draft, validation, writing – review and editing, formal analysis, data curation, supervision. **Frank Kyei‐Arthur**: conceptualization, methodology, formal analysis, visualization, writing – review and editing, writing – original draft, project administration, software, data curation, validation. **Vida Owusua Mensah**: methodology, investigation, conceptualization, formal analysis, validation, project administration, writing – review and editing, writing – original draft. **Godwin A. Kubi**: methodology, writing – original draft, writing – review and editing, visualization, validation, formal analysis. **Gloria Ayorkor Charway**: writing – original draft, writing – review and editing, validation, methodology, software, formal analysis, data curation. **Joseph Kwatsenu**: methodology, software, formal analysis, visualization, writing – review and editing. **Marian Kpakpah**: conceptualization, methodology, software, formal analysis, validation, visualization, writing – original draft, writing – review and editing, supervision. **Joha Issaka Braimah**: methodology, validation, formal analysis, software, data curation, supervision, writing – review and editing, writing – original draft. **Sylvester Kyei‐Gyamfi**: conceptualization, investigation, writing – original draft, methodology, validation, visualization, writing – review and editing, software, formal analysis, data curation, supervision. **Kelvin Aduful**: methodology, validation, visualization, writing – review and editing, formal analysis, software, data curation.

## Funding

The authors have nothing to report.

## Ethics Statement

The authors have nothing to report.

## Conflicts of Interest

The authors declare no conflicts of interest.

## Data Availability

This article is based exclusively on secondary data sources, including peer‐reviewed journal articles, national crash statistics and publicly accessible institutional and policy reports. All materials included in the systematic review are cited in the reference list and are accessible through academic databases or official organizational repositories.
